# Sports Nutrition Knowledge and Carbohydrate Intake in Young Male Elite Football Players: Insights from a Case Study of HNK Hajduk Academy

**DOI:** 10.3390/jfmk10020169

**Published:** 2025-05-11

**Authors:** Marta Tomljanovic, Ana Kezic, Mario Tomljanovic, Daniela Čačić Kenjerić

**Affiliations:** 1 Faculty of Kinesiology, University of Split, 21000 Split, Croatia; tomljanovicmarta7@gmail.com (M.T.); mario.tomljanovic@kifst.eu (M.T.); 2 Faculty of Food Technology, University of Osijek, 31000 Osijek, Croatia; daniela.kenjeric@ptfos.hr

**Keywords:** Mediterranean diet, carbohydrates, football performance, dietary adherence, endurance, young athletes

## Abstract

**Background:** Proper nutrition is crucial for optimizing performance and recovery in elite young football players. This study aimed to assess sports nutrition knowledge, adherence to the Mediterranean diet (MD), and carbohydrate intake across different training phases, evaluating their relations with match performance. **Methods:** Thirty-three male HNK Hajduk academy players (15–19 years) completed a nutrition questionnaire and a seven-day food diary, while performance metrics were analyzed using GPS tracking. **Results:** The results showed that sports nutrition knowledge was generally low (43.0 ± 17.0%), with significant misconceptions about carbohydrate fueling strategies. Players significantly overestimated their MD adherence, with a self-reported KIDMED index (6.06 ± 2.41) notably higher than the corrected score derived from food diaries (4.21 ± 2.53, *p* < 0.001). Carbohydrate intake was suboptimal on match-related days (3.64 g/kg on match day, 4.45 g/kg on the day before), failing to meet the recommended minimum of 6 g/kg per day. Regression analysis predicted that energy (β = 0.83; *p* = 0.02) and carbohydrate intake (β = 0.69; *p* = 0.03) on match day significantly predicted distance covered per minute, with a positive relationship observed for both outcomes, highlighting its impact on endurance. However, no significant relationship was found between carbohydrate intake and maximum sprint speed. **Conclusions:** These findings underscore the need for structured nutrition education within football academies to enhance players’ dietary habits and performance outcomes. Future research should focus on longitudinal interventions to assess how improved nutrition knowledge influences dietary adherence and athletic performance over time. Although knowledge of sports nutrition is fundamental, practical training and education to improve dietary competencies are crucial for athletes to effectively apply this knowledge in daily training and match preparations.

## 1. Introduction

Proper nutrition is crucial for optimal football performance, influencing both players’ physical capabilities and their ability to recover and maintain health [1]. The high-intensity, intermittent nature of football requires tailored dietary strategies that account for the specific physiological demands of the sport. Elite male players, for instance, execute around 36 sprints per game at speeds exceeding 21 km/h, with an average recovery time of 195 s between sprints [2]. Compared to recreational players, elite athletes demonstrate superior physical and physiological traits such as maximal isometric force, vertical jump height, and faster sprint times [3].

To meet the nutritional demands of elite athletes, a ‘food first’ approach is essential, emphasizing whole foods over supplements. Meeting these demands is crucial for supporting training, promoting growth, and preventing injuries, particularly for young athletes [4]. Despite the growing emphasis on sports nutrition, research shows that many young athletes have inadequate knowledge of proper dietary practices, which can negatively impact their performance and long-term health outcomes [5]. Key gaps in their nutrition knowledge include an understanding of macronutrient and micronutrient requirements, hydration strategies, and nutrient timing to maximize energy and recovery [6]. Experts recommend that the daily energy intake for football players ranges from 2500 to 3500 kcal, with macronutrient distribution typically consisting of 55–65% carbohydrates, 12–15% protein, and less than 30% fat [7,8]. Carbohydrate consumption should be around 8 g/kg of body mass per day, with hydration strategies involving 500 mL before a match and another 500 mL at halftime [9]. According to Burke et al. [10], timing carbohydrate consumption before, during, and after exercise is essential to maintain high carbohydrate availability during intense physical activity. For sustained high-intensity activities lasting about 1 h, even small amounts of carbohydrate can improve performance through effects on the central nervous system [11]. Despite these recommendations, studies show that young football players often rely on coaches, peers, and parents for nutrition advice, rather than qualified professionals [12]. As a result, they demonstrate low to moderate levels of nutrition knowledge, with a better understanding of general nutrition than sports-specific topics [13,14,15]. Knowledge gaps, particularly in macronutrient timing, hydration, and fueling strategies for performance, hinder athletes from optimizing their nutrition. Studies from India, Libya, Poland, and Australia consistently highlight these knowledge deficiencies. In India, football players demonstrated inadequate knowledge of general nutrition and nutrient timing for competition [16]. A Libyan study found that only 22% of participants knew what foods to consume before and after exercise, and 81% failed to identify the proper nutrients for consumption during exercise [17]. Polish athletes exhibited poor knowledge of dietary supplements and general sports nutrition [18], while Australian players showed better understanding of macronutrients but lacked knowledge about supplements, micronutrients, and sports nutrition specifics [19]. These findings underline the need for targeted nutrition education programs to bridge these gaps. Formal education from qualified professionals has been shown to significantly increase athletes’ nutrition knowledge compared to informal sources like peers or coaches [17,19]. Such programs should focus on practical applications of nutrition, including nutrient timing, appropriate food choices before, during, and after competition, and the proper use of supplements. Personalized nutrition education tailored to the individual needs of athletes is crucial for enhancing their dietary habits and improving performance. Additionally, players’ nutrition behaviors are influenced by access to food, cultural beliefs, and personal preferences, making it important to address these factors when developing educational programs.

The Mediterranean diet (MD) has shown potential benefits for athletic performance, with studies suggesting that it may improve muscle endurance, power, and anaerobic performance [20,21] compared to standard Western diets that are mainly high in empty calories but low in nutrient density and dietary fiber. Characterized by a high consumption of fruits, vegetables, whole grains, legumes, nuts, and olive oil, the MD provides essential nutrients that support energy production, reduce inflammation, and promote cardiovascular health. Research indicates that adherence to the MD is associated with better endurance, improved recovery, and lower oxidative stress [22]. The Mediterranean Diet Quality Index for children and adolescents (KIDMED) has been used to assess adherence, with studies showing moderate to high adherence levels among young athletes, with scores typically ranging from 6.9 to 7.83 on a scale of 0 to 10 points [23,24,25,26,27,28]. However, self-reported dietary intake can be biased, and, thus, adherence should be complemented with detailed food diaries to provide a more accurate measure.

The existing literature suggests that youth academy players typically maintain consistent carbohydrate intake across match days, pre-match days, and rest days, although levels often fall short of recommendations (match day 6–8 g/kg; pre-match day 7–10 g/kg; rest day 3–5 g/kg), especially on match days and during intense training [29,30,31,32]. Carbohydrate intake significantly impacts performance in football, with higher carbohydrate diets improving running distance, sprint performance, and endurance [33,34]. Studies show that players consuming higher carbohydrate diets performed 33% more high-intensity exercise during matches [35]. GPS data have shown that carbohydrate loading can improve running outputs and metabolic efficiency [36].

This study seeks to bridge the gap in knowledge regarding sports nutrition by evaluating young football players’ adherence to the MD. The primary aim of this study is to evaluate sports nutrition knowledge, dietary habits, and carbohydrate intake in young male football players and to assess how these factors influence their match performance.

## 2. Materials and Methods

Participants were recruited from the male football academy HNK Hajduk, consisting of cadet and junior players (aged 15–19 years) who play in the 1st Croatian football league and the Al Abtal International Cup. A total of 33 players participated, including 9% goalkeepers, 36% defenders, 27% midfielders, and 27% forwards. Sociodemographic and anthropometric data, including average height (181.2 ± 8.4 cm), weight (72.4 ± 9.2 kg), BMI (20.2 ± 4.7 kg/m^2^), and training experience (7.5 ± 1.5 years), were collected for all participants. All players were actively involved in full training and competition, with a weekly schedule consisting of five training days, one match day, and one recovery day. Ethical approval was obtained from the University of Split (Class: 003-08/25-04/001, No. 2181-205-02-05-25-001), and informed consent was provided by both participants and their legal guardians.

The nutritional knowledge questionnaire was designed using previously validated questionnaires [37,38,39] (Supplementary Table S1). It consisted of three sections: (1) demographic information (14 questions), (2) nutrition knowledge (11 questions), and (3) KIDMED (16 questions). The nutrition knowledge section covered both general and sports nutrition, with questions adapted from previously validated questionnaires, including the ULTRA-Q [37] and GNKQ [38]. The adherence section was based on the KIDMED index, a concise and practical tool for assessing adherence to the MD in this group of respondents [39]. The questionnaire had been previously piloted in a football academy in Dalmatia across all selections (seniors, juniors, cadets, and pioneers). The sports nutrition questionnaire was administered for approximately 10 min, using Google Forms in the presence of researchers to minimize discussion of responses between participants. We ensured compliance with data security laws by using a secure version of Google Forms, with data stored in an encrypted format. The nutrition knowledge score was based on the number of correct responses, categorized as follows: “poor” (0–49%), “average” (50–65%), “good” (66–75%), and “excellent” (75–100%) [40].

Energy intake was assessed over a seven-day period during the competitive season using a self-reported weighed food diary and a 24 h dietary recall. This period was chosen to balance comprehensive dietary data with participant compliance, as recommended in previous studies [41,42]. Participants recorded their food intake, including consumption times, preparation methods, portion sizes, and brand names. Additional details, such as condiments or unspecified food items, were recorded as needed. Players primarily followed a combined dietary approach, with one main meal provided by the academy, while the remaining meals were at their discretion, either prepared by their families or consumed outside the academy. Energy intake was analyzed using Program Prehrane software (version 6.69.1, Croatia), and all dietary data were processed by a single researcher who also led the familiarization workshops, as recommended by Deakin et al. [43]. In addition to energy and carbohydrate intake, the KIDMED index was calculated based on the submitted food diaries to assess adherence to the MD.

Physical performance data were collected using GPS tracking systems (Catapult Sports, Melbourne, Australia), which measured key metrics such as speed, distance, and intensity. The analysis focused specifically on distance covered per minute and maximum sprint speed, which are indicators of physical performance and fatigue during matches and training. GPS data were collected from 25 players, excluding goalkeepers and those who did not participate in the match.

Data collection took place during the first half of the competitive season, from the last week of October through the first days of November. Participants first completed the online questionnaire and then followed with a seven-day dietary recording period. During this period, three key days were selected for monitoring carbohydrate intake: rest day (DAY OFF), the day before the match (MD-1), and match day (MATCH DAY). GPS data were recorded during the match to assess performance in relation to dietary intake. The match played during this study was held at home, eliminating potential confounding factors such as travel, climate changes, or limited access to standardized nutrition.

Statistical analyses included descriptive statistics (mean, standard deviation, minimum, and maximum values) for energy and carbohydrate intake, as well as GPS performance metrics. Independent *t*-tests were used to compare nutrition knowledge scores based on prior nutritional education, and dependent *t*-tests assessed differences between self-reported and calculated KIDMED indices. Repeated measures ANOVA was employed to examine differences in carbohydrate intake across the three key days, with pairwise comparisons adjusted using the Bonferroni correction. Multiple regression analysis assessed the relationship between carbohydrate intake on match day and the day before the match as predictors of GPS performance metrics. Statistical significance was set at *p* < 0.05. All statistical analyses were conducted using Statistica 14.1 (TIBCO Software Inc., San Ramon, CA, USA).

## 3. Results

The average score on the sports nutrition knowledge questionnaire was 43.0 ± 17.0%, with scores ranging from 0% to 70%. In terms of age, there was no significant difference between cadet and junior athletes (*p* = 0.39). Nevertheless, 75.7% of participants indicated having some prior nutritional knowledge, and their average score was notably higher compared to those without such knowledge (48.1% vs. 26.7%; *t* = 3.56, df = 31, *p* < 0.000). Athletes who had received nutritional guidance (63.6%) did not perform significantly differently from those who had not (45.0% vs. 40.2%; *t* = 0.77, df = 31, *p* = 0.450). However, individuals who were formally advised by a nutritionist (42.4%) demonstrated a significantly better performance on the questionnaire compared to those without such guidance (51.0% vs. 37.0%; *t* = −0.25, df = 31, *p* < 0.02). The most frequently incorrect questions (with fewer than 39% answering correctly) pertained to pre-game food selection, post-game meal timing, carbohydrate and water intake, as well as the quantity of proteins, carbohydrates, and fats in specific foods. More than 65% of participants correctly answered only the question concerning snack consumption during training.

The KIDMED index was assessed using two approaches. First, participants self-reported their adherence to the MD via the KIDMED questionnaire. Then, a calculated KIDMED index was derived from their 7-day dietary records, following the same scoring criteria but based on actual food intake. The difference between the two scores was analyzed with the use of a dependent sample *t*-test to determine the extent of misreporting or overestimation in dietary self-assessment. A significant difference was found (*t* = 6.54, *p* = 0.00) between the self-reported KIDMED index from the questionnaire (mean = 6.06) and the calculated KIDMED index based on the participants’ food diaries (mean = 4.21). Athletes generally reported a higher adherence to the MD compared to their actual dietary intake. The greatest discrepancies were observed in the consumption of fruits, vegetables, and dairy products, which were overreported in the questionnaire, while the intake of processed foods and sugary beverages was underreported.

**Figure 1:**
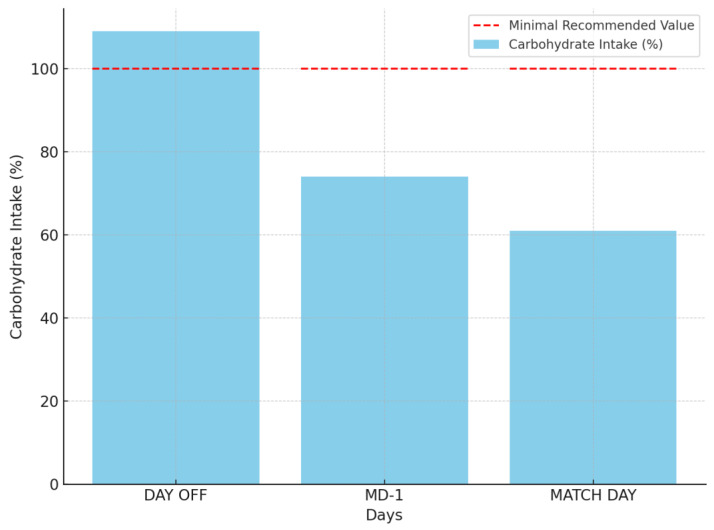
Average carbohydrate intake as a percentage of the minimal recommended intake across different training days (day off, day before the match (MD-1), and match day). The red dashed line indicates the minimal recommended carbohydrate intake.

The analysis of carbohydrate intake revealed suboptimal distribution across some training days (Table 1, Figure 1). On average, football players’ intake of carbohydrates on DAY OFF was 3.28 g/kg, 4.45 g/kg on MD-1, and 3.64 g/kg on MATCH DAY. In comparison to the recommended intake values per day (≥6 g/kg on pre-match and match days), it is evident that athletes failed to meet their carbohydrate requirements on the most critical days. Figure 1 illustrates that, while carbohydrate intake on rest days was above the recommended minimal value (≥3 g/kg), the intake on both pre-match and match days was significantly below the optimal threshold (75% and 61.7%). The failure to meet these recommendations may indicate insufficient glycogen storage before competition, potentially affecting endurance and recovery.

Regarding the CH intake in g/kg per day, the results of the repeated measures ANOVA showed a significant main effect of testing day, F(2, 64) = 155.42, *p* = 0.000, η^2^_p_ = 0.84, indicating that the dependent variable varied substantially across the rest day, the day before the match, and the match day. Pairwise comparisons with Bonferroni correction revealed that values at DAY OFF (M = 3.28, SD = 1.24) and MATCH DAY (M = 3.64, SD = 1.22) were significantly lower than at MD-1 (M = 4.45, SD = 1.74, *p* < 0.002), while the difference between DAY OFF and MATCH DAY was not significant (*p* = 0.19).

The regression analysis examined the relationship between energy intake and carbohydrate intake on the match day and the day before the match as predictors of distance covered (m/min) during the match (Table 2). The overall model was statistically significant (*p* < 0.05), indicating that dietary intake plays a role in match-day running performance. However, only energy intake and carbohydrate intake on the match day emerged as significant predictors (*p* < 0.05), while carbohydrate intake on the day before the match did not contribute significantly to the model. A separate regression analysis was conducted to examine whether energy intake and carbohydrate intake on the match day and the day before the match could predict maximum sprinting speed (km/h) recorded via GPS. The model was not statistically significant (*p* > 0.05), indicating that these nutritional variables did not meaningfully explain variations in maximum sprinting speed.

## 4. Discussion

This study aimed to evaluate sports nutrition knowledge, adherence to the MD, and carbohydrate intake among young male football players while assessing how these factors impact performance during match play. The findings highlight critical areas that require attention to optimize dietary practices and enhance athletic performance.

### 4.1. Sports Nutrition Knowledge

The study results revealed low overall levels of sports nutrition knowledge, with an average questionnaire score of 43.0% and notable misconceptions about carbohydrate intake, pre-game meal composition, and post-match recovery nutrition. Athletes who had received formal advice from a nutritionist performed significantly better on the nutrition questionnaire, emphasizing the importance of structured education. This finding aligns with previous research demonstrating that players with access to professional nutritional guidance make better dietary choices than those who rely on informal sources [44]. Despite the known impact of nutrition on performance, the lack of knowledge regarding carbohydrate fueling strategies is particularly concerning. Previous studies have demonstrated that low carbohydrate literacy among athletes often leads to inadequate glycogen replenishment, which can impair high-intensity efforts [45,46]. Given these knowledge gaps, there is a strong need for targeted education programs within football academies, ensuring that players, coaches, and staff understand the critical role of pre-match carbohydrate loading and post-match recovery nutrition. To effectively implement carbohydrate periodization strategies, athletes must not only receive practical guidance but also understand the physiological rationale behind these approaches to maximize adherence and performance benefits [4].

### 4.2. Discrepancies Between Self-Reported and Actual Mediterranean Diet Adherence

The findings also indicate a significant overestimation of adherence to the MD when comparing self-reported KIDMED scores to actual food intake recorded in dietary diaries. Players tended to overreport their consumption of fruits, vegetables, and dairy while underreporting processed foods and sugary beverages [47]. This discrepancy highlights the well-documented issue of social desirability bias in dietary self-reporting [26]. Interestingly, the corrected KIDMED scores in this study were lower than those reported in previous research on young football players. For instance, Leão et al. [48] found an average KIDMED score of 8.36 among Portuguese youth players, which is significantly higher than the present study’s corrected score of 4.21. These findings suggest that adherence to the MD may vary based on cultural and regional dietary habits, as well as access to MD-friendly foods. Since previous research has linked higher MD adherence to improved endurance, faster recovery, and lower inflammation markers [22,49], the lower-than-expected adherence observed in this study may have important performance implications. While the MD provides an excellent foundation for overall health, cardiovascular function, and recovery, it may not sufficiently address the specific energy demands of high-intensity, intermittent sports like football, especially for fast and explosive positions such as wingers and forwards. Contemporary sports nutrition emphasizes the importance of carbohydrate periodization and nutrient timing to align energy intake with the physical demands of training and competition, a practice commonly implemented by elite clubs like Liverpool FC [50]. These strategies—such as increasing carbohydrate availability on match days while reducing intake during lower-load sessions—optimize glycogen stores and performance [4]. Additionally, individualized dietary modifications, including low-FODMAP and gluten-free approaches, are increasingly used to manage gastrointestinal symptoms in sensitive athletes [1,51]. Such targeted interventions have been shown to reduce digestive discomfort and enhance both training quality and overall performance. While the MD supports endurance and recovery, optimal football performance may require more sport-specific and position-specific nutritional strategies, which should also be included in educational programs to address the unique needs of football players.

### 4.3. Carbohydrate Intake and Performance: A Critical Gap in Fueling Strategies

One of the most important findings of this study is the inadequate carbohydrate intake on match-related days. Players consumed 3.28 g/kg on a rest day, 4.45 g/kg on the day before the match, and 3.64 g/kg on the match day, all of which are below the recommended intake of 6–8 g/kg per day [4]. This deficiency suggests that players are not adequately fueling to optimize glycogen stores before competition, which may impact their ability to sustain high-intensity efforts during matches. The GPS performance analysis supports this concern, showing that players with higher carbohydrate intake on match day covered significantly more distance per minute. This aligns with previous studies demonstrating that higher carbohydrate availability is directly linked to greater running capacity and reduced fatigue in football players [52,53]. Notably, players who consumed closer to or above 6 g/kg on match day exhibited greater endurance, reinforcing the well-established relationship between glycogen availability and prolonged high-intensity activity [35]. However, this study found that carbohydrate intake had a weaker association with maximum sprint speed, suggesting that, while endurance performance is carbohydrate-dependent, sprint capacity may be more influenced by neuromuscular and anaerobic factors [54]. These findings indicate that players need not only to optimize carbohydrate intake but also ensure that strength and sprint-specific training is adequately incorporated to enhance short-duration, high-intensity efforts.

### 4.4. Practical Implications for Football Academies

The findings of this study emphasize the importance of structured nutrition education, optimized carbohydrate intake, and dietary adherence for young football players. Given the observed knowledge gaps, football academies should prioritize formal nutrition education programs that go beyond general dietary advice and focus on practical, sport-specific strategies. Providing players with hands-on workshops where they can learn to plan their own match-day meals, understand food labels, and recognize the importance of nutrient timing could significantly improve dietary habits. Ensuring that players meet their carbohydrate needs on match-related days should be a key priority for clubs. Many players in this study failed to consume the recommended 6–8 g/kg/day of carbohydrates before competition, which may have negatively impacted their endurance and overall match performance, as has been observed in previous studies [55,56]. To address this, pre-match carbohydrate loading strategies should be encouraged, focusing on high-quality carbohydrate sources such as pasta, rice, potatoes, and fruit-based options. Providing pre-prepared, nutrient-optimized meals within academy facilities could further support players in achieving these targets, reducing reliance on suboptimal or inconsistent individual choices.

Additionally, given that players overestimated their adherence to the MD, it is essential to implement regular dietary assessments to ensure that actual intake aligns with recommended nutritional guidelines. This could involve a combination of food diaries, guided recalls, and nutritionist-led evaluations, helping players recognize potential gaps in their diets and make necessary adjustments. Standardizing meal plans within academy settings, particularly for critical training and competition days, would also help to bridge the gap between perceived and actual dietary behaviors.

Ultimately, football academies must take an active role in shaping young athletes’ nutritional habits, ensuring that players not only understand the importance of proper fueling but also have the resources, education, and structured support to implement these strategies effectively. By embedding nutrition literacy and meal planning into daily routines, clubs can help players optimize their performance, recovery, and long-term health, setting the foundation for sustainable dietary habits that will benefit them throughout their careers.

### 4.5. Study Limitations and Future Research Directions

While this study provides valuable insights into the nutritional habits and performance of young elite football players, certain limitations should be acknowledged. Self-reported dietary intake, despite validation efforts such as 24 h recall interviews and portion size estimation support, remains inherently prone to bias. Players may have unintentionally misreported their food intake due to recall errors or social desirability bias, particularly when assessing their adherence to the MD. Future research should consider integrating biomarker analyses, such as glycogen muscle biopsies or blood glucose monitoring, to obtain a more objective assessment of actual dietary adherence. Also, while Google Forms provides a convenient platform for data collection, it may not fully align with the highest standards of data security regulations.

Another limitation lies in the specific sample used in this study, as all participants were drawn from a single football academy. While the findings provide a valuable case study, they may not be fully generalized to other academies, leagues, or cultural contexts, where dietary habits, food availability, and training methodologies might differ. Expanding this research across multiple clubs and regions would provide a more comprehensive understanding of how nutritional behaviors vary across different footballing environments.

Additionally, while GPS tracking provided an objective measure of distance covered and sprinting ability, this study did not incorporate physiological markers of fatigue or metabolic efficiency, such as lactate thresholds or muscle glycogen depletion rates. Including these measures in future studies could offer a deeper physiological understanding of how dietary intake directly influences endurance and recovery.

Another important area that warrants further investigation is hydration status and its impact on match performance. Future research should assess fluid intake habits, sweat rates, and electrolyte balance in young athletes, especially in warm climates or during intense training periods. By integrating hydration monitoring methods, such as urine-specific gravity testing or body mass fluctuations, researchers could gain deeper insights into how hydration strategies influence endurance, fatigue levels, and injury prevention in football players.

This study focused solely on male athletes. Future research should include female athletes to compare dietary behaviors and performance outcomes across genders. Also, further studies should consider the inclusion of phase angle measurements as an objective biomarker of nutritional status, which may offer additional insights into the relationship between dietary habits and athletes’ physical health.

Finally, this research represents a snapshot of dietary and performance data over a short time frame, meaning that it does not account for long-term adaptations resulting from sustained changes in nutrition. Given that training cycles, opponent variability, and various internal and external factors (such as training load, motivation, and external stressors) can significantly influence both diet and performance, a longitudinal study design would be necessary to better capture typical patterns and outcomes over time. Future research should explore longitudinal interventions, tracking dietary modifications over an entire season to assess whether consistent improvements in carbohydrate intake and MD adherence translate into measurable performance gains over time. Also, tailoring carbohydrate strategies based on positional roles could enhance both practical relevance and nutritional recommendations.

## 5. Conclusions

The findings of this study emphasize the importance of structured nutrition education, accurate dietary self-assessment, and optimized carbohydrate intake for young football players. While formal nutrition guidance improved knowledge, overall scores remained low, highlighting the need for targeted interventions. Athletes’ self-reported adherence to the MD was overestimated, suggesting that objective dietary assessments should be incorporated into nutritional programs. Finally, carbohydrate intake was inadequate on match days, which may have negatively impacted endurance performance. However, this was an observational study, which suggests a relationship between carbohydrate intake and endurance performance, while a randomized controlled intervention—where one group receives a structured, high-carbohydrate meal plan and another follows their usual diet—would provide stronger evidence that dietary intake directly impacts performance outcomes. Future research should explore strategies to improve athletes’ dietary practices and assess their direct impact on match performance.

## Figures and Tables

**Table 1:** Descriptive statistics on energy (E (kcal)) and carbohydrate (CH (g), CH (g/kg)) intake during three distinctive days (rest day (DAY OFF), the day before the match (MD-1) and MATCH DAY), as well as GPS data on covered distance and maximal speed; results of *t*-test for dependent samples between carbohydrate intake (g/kg) of athletes and recommended values per day (^a, b^).

	Mean	SD	Min	Max	Recommended Values for Age/Sport
DAY OFF—E (kcal)	2352.77	376.99	1629.40	3237.30	
DAY OFF—CH (g)	252.68	57.59	113.00	383.00	
DAY OFF—CH (g/kg)	3.28 ^a^	1.24	0.00	5.72	3–5 g/kg/day
MD-1—E (kcal)	2776.00	897.64	453.40	4956.90	
MD-1—CH (g)	315.14	114.12	45.10	642.00	
MD-1—CH (g/kg)	4.45 ^b^	1.74	0.73	10.52	6–8 g/kg/day
MATCH DAY—E (kcal)	2273.88	637.24	941.60	4104.90	
MATCH DAY—CH (g)	261.19	98.53	91.90	611.50	
MATCH DAY—CH (g/kg)	3.64 ^b^	1.22	1.31	7.55	6–8 g/kg/day
GPS distance (m/min)	108.02	9.73	93.65	130.40	
GPS max speed (km/h)	28.18	1.68	25.13	31.82	

Legend: ^a^—not significantly different than recommended values (*p* > 0.05), ^b^—significantly different than recommended values (*p* < 0.00).

**Table 2:** Results of regression analyses with energy and carbohydrate intake on different days as predictor variables and GPS data as criterion variables.

	GPS Distance (m/min)		GPS Max Speed (km/h)	
	Beta	*p*-Value	Beta	*p*-Value
MATCH DAY—E (kcal)	0.83	0.02	0.08	0.83
MATCH DAY—CH (g/kg)	0.69	0.03	0.19	0.59
MD-1—E (kcal)	0.18	0.62	0.32	0.44
MD-1—CH (g/kg)	0.29	0.41	0.45	0.27
	R = 0.59 R^2^ = 0.38 *p* = 0.05		R = 0.29 R^2^ = 0.10 *p* = 0.77	

## Data Availability

The data presented in this study are available on request from the corresponding author.

## Supplementary Materials

The following supporting information can be downloaded at: https://www.mdpi.com/article/10.3390/jfmk10020169/s1, Table S1: The nutritional knowledge questionnaire designed using previously validated questionnaires.

## Abbreviations

The following abbreviations are used in this manuscript:

BMI	Body Mass Index
KIDMED	Mediterranean Diet Quality Index for children and adolescents
MD	Mediterranean diet

## References

[1] Thomas D.T., Erdman K.A., Burke L.M. (2016). Position of the Academy of Nutrition and Dietetics, Dietitians of Canada, and the American College of Sports Medicine: Nutrition and athletic performance. J. Acad. Nutr. Diet..

[2] Bangsbo J., Mohr M. (2005). Variations In Running Speeds And Recovery Time After A Sprint During Top-class Soccer Matches. Med. Sci. Sports Exerc..

[3] Gissis I., Papadopoulos C., Kalapotharakos V.I., Sotiropoulos A., Komsis G., Manolopoulos E. (2006). Strength and speed characteristics of elite, subelite, and recreational young soccer players. Res. Sports Med..

[4] Burke L.M., Hawley J.A., Jeukendrup A., Morton J.P., Stellingwerff T., Maughan R.J. (2021). Toward a common understanding of diet–exercise strategies to manipulate fuel availability for training and competition preparation in endurance sport. Int. J. Sport. Nutr. Exerc. Metab..

[5] Heaney S., O’Connor H., Michael S., Gifford J., Naughton G. (2011). Nutrition knowledge in athletes: A systematic review. Int. J. Sport Nutr. Exerc. Metab..

[6] Jihad H.M. (2024). Nutrition for Football Players: Fueling Performance on the Field. Am. J. Soc. Humanit. Res..

[7] Martinho D.V., Naughton R.J., Leão C., Lemos J., Field A., Faria A., Sarmento H. (2023). Dietary intakes and daily distribution patterns of macronutrients in youth soccer players. Front. Nutr..

[8] Clark K. (1994). Nutritional guidance to soccer players for training and competition. J. Sport. Sci..

[9] Shephard R.J. (1990). Meeting carbohydrate and fluid needs in soccer. Can. J. Sport. Sci..

[10] Burke L.M., Hawley J.A., Wong S.H.S., Jeukendrup A.E. (2011). Carbohydrates for training and competition. J. Sport. Sci..

[11] Abreu R., Figueiredo P., Beckert P., Marques J.P., Amorim S., Caetano C., Carvalho P., Sá C., Cotovio R., Cruz J. (2021). Portuguese Football Federation consensus statement 2020: Nutrition and performance in football. BMJ Open Sport Exerc. Med..

[12] Devlin B.L., Belski R. (2015). Exploring General and Sports Nutrition and Food Knowledge in Elite Male Australian Athletes. Int. J. Sport Nutr. Exerc. Metab..

[13] Ray S., Mounce C.D., Gonzalez-Rodenas J., Prieto M.S., Brannan R. (2019). Assessment of a Nutrition Intervention on the Nutrition Knowledge of Adolescent Soccer Academy Players. Curr. Dev. Nutr..

[14] Noronha D.C., Santos M.I., Santos A.A., Corrente L.G., Fernandes R.K., Barreto A.C., Santos R.G., Santos R.S., Gomes L.P., Nascimento M.V. (2020). Nutrition Knowledge is Correlated with a Better Dietary Intake in Adolescent Soccer Players: A Cross-Sectional Study. J. Nutr. Metab..

[15] Chen Y., Sun Y., Liu Z., Hu D. (2022). Study on nutritional knowledge, attitude and behavior of Chinese school football players. Children.

[16] Subba A., Rai S. (2022). Exploring the level of awareness about sports nutrition knowledge among football players belonging to different ethnic groups of North Bengal, India. Res. Rev. Int. J. Multidiscip..

[17] Denna I., Elmabsout A., Eltuhami A., Alagory S., Alfirjani T., Barakat F., Almajouk S.A., Younis M. (2018). Evaluation of nutrition knowledge of professional football players. Ibnosina J. Med. Biomed. Sci..

[18] Staśkiewicz W., Grochowska-Niedworok E., Zydek G., Białek-Dratwa A., Grajek M., Jaruga-Sȩkowska S., Kowalski O., Kardas M. (2022). Changes in body composition during the macrocycle of professional football players in relation to sports nutrition knowledge. Front. Nutr..

[19] Trakman G.L., Forsyth A., Middleton K., Hoye R., Jenner S., Keenan S., Belski R. (2018). Australian football athletes lack awareness of current sport nutrition guidelines. Int. J. Sport Nutr. Exerc. Metab..

[20] Bach-Faig A., Berry E.M., Lairon D., Reguant J., Trichopoulou A., Dernini S., Medina F.X., Battino M., Belahsen R., Miranda G. (2011). Mediterranean diet pyramid today. Public Health Nutr..

[21] Shen J., Wilmot K.A., Ghasemzadeh N., Molloy D.L., Burkman G., Mekonnen G., Sperling L.S. (2015). Mediterranean dietary patterns and cardiovascular health. Annu. Rev. Nutr..

[22] Sureda A., Bibiloni M.D.M., Julibert A., Bouzas C., Argelich E., Llompart I., Tur J.A. (2014). Adherence to the Mediterranean diet and inflammatory markers. Nutrients.

[23] Cabrera S.G., Fernández N.H., Hernández C.R., Nissensohn M., Román-Viñas B., Serra-Majem L. (2015). KIDMED test; prevalence of low adherence to the Mediterranean Diet in children and young; a systematic review. Nutr. Hosp..

[24] Moreno C., Pasquarelli B.N., Romaguera D., Martínez S., Tauler P.J., Aguiló A. (2013). Perfil nutricional de jovens jogadores de futebol da cidade de Palma de Mallorca, España. Rev. Bras. Futebol..

[25] López Secanell I., Rico Mateu R. (2019). La adhesión a la dieta mediterránea en los jugadores de las categorías inferiores de un club de futbol de alto rendimiento y su relación con el índice de masa corporal. TRANCES.

[26] Manzano-Carrasco S., Felipe J.L., Sanchez-Sanchez J., Hernandez-Martin A., Gallardo L., Garcia-Unanue J. (2020). Physical fitness, body composition, and adherence to the Mediterranean diet in young football players: Influence of the 20 mSRT score and maturational stage. Int. J. Environ. Res. Public Health.

[27] del Mar Fernández-Álvarez M., Martín-Payo R., Zabaleta-del-Olmo E., García-García R., Cuesta M., Gonzalez-Méndez X. (2020). Assessment of diet quality and physical activity of soccer players aged 13 to 16, from the Principality of Asturias, Spain. An. Pediatr. (Engl. Ed.).

[28] Santos-Sánchez G., Cruz-Chamorro I., Perza-Castillo J.L., Vicente-Salar N. (2021). Body Composition Assessment and Mediterranean Diet Adherence in U12 Spanish Male Professional Soccer Players: Cross-Sectional Study. Nutrients.

[29] Briggs M.A., Cockburn E., Rumbold P.L., Rae G., Stevenson E.J., Russell M. (2015). Assessment of energy intake and energy expenditure of male adolescent academy-level soccer players during a competitive week. Nutrients.

[30] Naughton R.J., Drust B., O’Boyle A., Morgans R., Abayomi J., Davies I.G., Mahon E. (2016). Daily distribution of carbohydrate, protein and fat intake in elite youth academy soccer players over a 7-day training period. Int. J. Sport Nutr. Exerc. Metab..

[31] Carter J.L., Lee D.J., Fenner J.S., Ranchordas M.K., Cole M. (2024). Contemporary educational and behavior change strategies improve dietary practices around a match in professional soccer players. J. Int. Soc. Sports Nutr..

[32] Stables R.G., Hannon M.P., Costello N.B., McHaffie S.J., Sodhi J.S., Close G.L., Morton J.P. (2024). Acute fuelling and recovery practices of academy soccer players: Implications for growth, maturation, and physical performance. Sci. Med. Football.

[33] Kazemi A., Racil G., Ahmadi H., Amir H., Moghadam M.B., Karami P., Henselman M. (2023). Improved physical performance of elite soccer players based on GPS results after 4 days of carbohydrate loading followed by 3 days of low carbohydrate diet. J. Int. Soc. Sports Nutr..

[34] O’Brien L., Collins K., Amirabdollhian F. (2021). Exploring Sports Nutrition Knowledge in Elite Gaelic Footballers. Nutrients.

[35] Balsom P.D., Gaitanos G.C., Söderlund K., Ekblom B. (1999). High-intensity exercise and muscle glycogen availability in humans. Acta Physiol. Scand..

[36] Routledge H., Graham S., Di Michele R., Burgess D., Erskine R., Close G., Morton J. (2020). Training Load and Carbohydrate Periodization Practices of Elite Male Australian Football Players: Evidence of Fueling for the Work Required. Int. J. Sport Nutr. Exerc. Metab..

[37] Blennerhassett C., McNaughton L.R., Cronin L., Sparks S.A. (2018). Development and implementation of a nutrition knowledge questionnaire for ultra-endurance athletes. Int. J. Sport Nutr. Exerc. Metab..

[38] Parmenter K., Wardle J. (1999). Development of a general nutrition knowledge questionnaire for adults. Eur. J. Clin. Nutr..

[39] Serra-Majem L., Ribas L., Ngo J., Ortega R.M., García A., Pérez-Rodrigo C., Aranceta J. (2004). Food, youth, and the Mediterranean diet in Spain. Development of KIDMED, Mediterranean Diet Quality Index in children and adolescents. Public Health Nutr..

[40] Jenner S.L., Trakman G., Coutts A., Kempton T., Ryan S., Forsyth A., Belski R. (2018). Dietary intake of professional Australian football athletes surrounding body composition assessment. J. Int. Soc. Sports Nutr..

[41] Russell M., Pennock A. (2011). Dietary analysis of young professional soccer players in 1 week during the competitive season. J. Strength Cond. Res..

[42] Caccialanza R., Cameletti B., Cavallaro G. (2007). Nutritional intake of young Italian high-level soccer players: Under-reporting is the essential outcome. J. Sports Sci. Med..

[43] Deakin V., Burke L., Deakin V. (2009). Measuring nutritional status of athletes: Clinical and research perspectives. Clinical Sports Nutrition.

[44] Hasanpouri A., Rahmani B., Gharakhanlou B.J., Solaimanian S., Shahsavari S., Rasouli A., Shiri-Shahsavar M.R. (2023). Nutritional knowledge, attitude, and practice of professional athletes in an Iranian population (a cross-sectional study). BMC Sports Sci. Med. Rehabil..

[45] Spronk I., Heaney S.E., Prvan T., O’Connor H.T. (2015). Relationship between general nutrition knowledge and dietary quality in elite athletes: A systematic review. Int. J. Sport Nutr. Exerc. Metab..

[46] Spendlove J.K., Heaney S., Gifford J.A., Prvan T., O’Connor H.T. (2018). Nutrition knowledge and dietary intake in adolescent elite athletes: A systematic review. Int. J. Sport Nutr. Exerc. Metab..

[47] Janiczak A., Devlin B.L., Forsyth A., Trakman G.L. (2022). A systematic review update of athletes’ nutrition knowledge and association with dietary intake. Br. J. Nutr..

[48] Leão C., Pinto J., Silva M. (2023). Adherence to the Mediterranean diet in young male soccer players. BMC Sports Sci. Med. Rehabil..

[49] Fernández-Ruiz V., Cámara M., Gómez-Martínez S. (2020). Adherence to dietary guidelines among Spanish adolescent athletes: Differences by gender and level of competition. Nutrients.

[50] Routledge H.E., James L.J., George T., Close G.L., Morton J.P. (2020). Periodic carbohydrate restriction and carbohydrate loading: Practical considerations for elite athletes. Sport Med..

[51] Lis D.M., Stellingwerff T., Shing C.M., Ahuja K.D., Fell J.W. (2019). Exploring the popularity, experiences, and beliefs surrounding gluten-free diets in nonceliac athletes. Int. J. Sport. Nutr. Exerc. Metab..

[52] Foskett A., Williams C., Boobis L., Tsintzas K. (2008). Carbohydrate availability and muscle energy metabolism during intermittent running. Med. Sci. Sports Exerc..

[53] Williams C., Rollo I. (2015). Carbohydrate nutrition and team sport performance. Sports Med..

[54] Mata F., Valenzuela P.L., Gimenez J., Tur C., Ferreria D., Domínguez R., Sanchez-Oliver A.J., Martínez Sanz J.M. (2019). Carbohydrate availability and physical performance: Physiological overview and practical recommendations. Nutrients.

[55] Cassidy C., Collins K., Shortall M. (2018). The precompetition macronutrient intake of elite Gaelic football players. Int. J. Sport. Nutr. Exerc. Metab..

[56] Pueyo M., Llodio I., Cámara J., Castillo D., Granados C. (2024). Influence of Carbohydrate Intake on Different Parameters of Soccer Players’ Performance: Systematic Review. Nutrients.

