# Supplementary material for: Sports Nutrition Knowledge and Carbohydrate Intake in Young Male Elite Football Players: Insights from a Case Study of HNK Hajduk Academy
Source: J Funct Morphol Kinesiol. 2025 May 11;10(2):169. doi: 10.3390/jfmk10020169 (PMC12101181; doi:10.3390/jfmk10020169)
Supplement: Supplementary file 1 [file jfmk-10-00169-s001.zip › jfmk-3540915-supplementary.pdf]

Assessment of nutrition knowledge, dietary attitudes and habits of football players

This questionnaire aims to check the knowledge of soccer players about nutrition in sports, nutritional attitudes and habits that soccer players implement in their daily lives.

* Označava obavezno pitanje

1. E-pošta *

____

Socio-demographic questions

2. Please state your name and surname:

____

3. How old are you? *

Označite samo jedan oval.

- ( ) <10 years old
- ( ) 10-14 years old
- ( ) 15-18 years old
- ( ) >18 years old

4. What subgroup do you fall into? *

Označite samo jedan oval.

- Pioneers
- Cadets
- Juniors
- Seniors

5. Which football position do you play? *

Označite samo jedan oval.

- Goalkeeper
- Defensive player
- Midfielder
- Forward

6. What is your education level? *

Označite samo jedan oval.

- Still in elementary school
- Finished elementary school
- Finished high school
- College degree

7. How many days a week do you train? *

Označite samo jedan oval.

- ≤3
- 4-5
- ≥6

8. Are you paid to play football? *

Označite samo jedan oval.

- Yes
- No

9. How long are you playing football *

Označite samo jedan oval.

- Less then 3 years
- 3-5 years
- More than 5 years

10. Do you have any nutrition knowledge? *

Označite samo jedan oval.

- Yes
- No

11. Have you ever gotten a nutrition advice? *

Označite samo jedan oval.

- Yes
- No

12. Do you work with a nutritionist? *

Označite samo jedan oval.

- Yes
- No

13. Are you on special nutritional regime? *

Označite samo jedan oval.

- Yes
- No

14. If your previous answer was yes, please state what regime is that?

____

KIDMED score

15. Do you eat one fruit or drink one fruit juice every day? *

Označite samo jedan oval.

- Yes
- No

16. Do you eat more than one fruit or drink more than one fruit juice every day? *

Označite samo jedan oval.

- Yes
- No

17. Do you eat fresh or cooked vegetables regularly once a day? *

Označite samo jedan oval.

- Yes
- No

18. Do you eat fresh or cooked vegetables more than once a day? *

Označite samo jedan oval.

- Yes
- No

19. Do you consume fish regularly? (at least 2-3/week) *

Označite samo jedan oval.

- Yes
- No

20. Do you go to fast food >1/week? *

Označite samo jedan oval.

- Yes
- No

21. Do you eat pulses >1/week? *

Označite samo jedan oval.

- Yes
- No

22. Do you consume pasta or rice almost every day (5 or more per week)? *

Označite samo jedan oval.

- Yes
- No

23. Do you have cereals or grains (bread, etc) for breakfast? *

Označite samo jedan oval.

- Yes
- No

24. Do you consume nuts regularly (at least 2-3/week)? *

Označite samo jedan oval.

- Yes
- No

25. Do you use olive oil at home? *

Označite samo jedan oval.

- Yes
- No

26. Do you skip breakfast? *

Označite samo jedan oval.

- Yes
- No

27. Do you have a dairy product for breakfast (yogurt, milk, etc)? *

Označite samo jedan oval.

- Yes
- No

28. Do you have a commercially baked goods or pastries for breakfast? *

Označite samo jedan oval.

- Yes
- No

29. Do you take two yogurts and/or some cheese (40g) daily? *

Označite samo jedan oval.

- Yes
- No

30. Do you take sweets and candy several times every day? *

Označite samo jedan oval.

- Yes
- No

Nutritional knowledge and attitudes assessment

31. Soccer players should: *

Označite samo jedan oval.

- Drink 50-100 mL of liquid every 15-20 minutes
- Suck ice cubes rather then drink liquids during workout
- Drink sports drinks instead of water during workout
- Drink according to hydration plan based on weight changes
- Not sure

32. Before the match or football practice, players should eat foods rich in: *

Označite samo jedan oval.

- Liquids, fats and carbohydrates
- Liquids, fiber and carbohydrates
- Liquids and carbohydrates
- Not sure

33. During the game or practice, players should focus on: *

Označite samo jedan oval.

- Liquids, fiber and fats
- Liquids and proteins
- Liquids and carbohydrates
- Not sure

34. Best snack for intensive 90-minute workout: *

Označite samo jedan oval.

- Protein shake
- Ripe banana
- Two boiled eggs
- Fistfull of nuts
- Not sure

35. After football practice or game, players should consume a meal rich in: *

Označite samo jedan oval.

- Proteins, fats and carbohydrates
- Just proteins
- Just carbohydrates
- Carbohydrates and proteins
- Not sure

36. Most important nutrient that is necessary for replenishing after football practice is: *

Označite samo jedan oval.

- Carbohydrates
- Proteins
- Fats
- Not sure

37. When a player is training daily, optimal time frame for food consumation is: *

Označite samo jedan oval.

- Between 2-3 hours
- Within 1 hour
- Within 30 minutes
- Not sure

38. Do you agree with the following statemets: *

Označite samo jedan oval po retku.

	I completely agree	I agree	I don't agree nor disagree	I disagree	I completely disagree
**Eating carbohydrates during exercise makes it harder to build strength and muscle.**					
**Eating carbohydrates during exercise will help keep blood sugar levels stable.**					
**A high-carbohydrate diet helps reduce muscle breakdown in the body.**					
**Energy drinks such as 'Red Bull' are good drinks for 30 minutes before exercise.**					
**Fruit juice is a good liquid during training.**					
**The best advice for a player about fluids during training would [illegible]**					

be to drink when thirsty.

A fluid loss of as little as 2% of an athlete's body weight can reduce endurance performance, especially in the heat.

Proteins are the main fuel for muscles during exercise.

If you eat more energy from protein than you need, you can gain weight.

Eating more protein is the most important change in your diet if you want to gain more muscle.

The body has a limited ability to use protein for muscle protein synthesis.

39. Choose if the following groceries are rich or poor in proteins: *

Označite samo jedan oval po retku.

	Rich	Poor	Don't know
**Turkey**			
**Baked beans**			
**Fruit**			
**Butter**			
**Cornflakes**			
**Peanuts**			

40. Choose if the following groceries are rich or poor in carbohydrates: *

Označite samo jedan oval po retku.

	Rich	Poor	Don't know
**Chicken**			
**Baked beans**			
**White bread**			
**Butter**			
**Cornflakes**			
**Rice pudding**			

41. Choose if the following groceries are rich or poor in fats: *

Označite samo jedan oval po retku.

	Rich	Poor	Don't know
**Avocado**	⬭	⬭	⬭
**Baked beans**	⬭	⬭	⬭
**Pasta**	⬭	⬭	⬭
**Butter**	⬭	⬭	⬭
**Cottage cheese**	⬭	⬭	⬭
**Rice pudding**	⬭	⬭	⬭
**Peanuts**	⬭	⬭	⬭
**White bread**	⬭	⬭	⬭
**Honey**	⬭	⬭	⬭
**Cheddar cheese**	⬭	⬭	⬭

Consent

By clicking on the option "I accept that my answers are used for the purpose of validating the questionnaire for the doctoral dissertation", you accept that your answers are used for the further purpose of improving and validating the questionnaire.

42. Consent *

Označite samo jedan oval.

- ⬭ I accept that my answers to be used for the purpose of validating the questionnaire for the doctoral dissertation
- ⬭ I don't accept that my answers to be used for the purpose of validating the questionnaire for the doctoral dissertation

Google nije izradio niti podržava ovaj sadržaj.

Google Obrasci
